# Assessing the Competition between Bulk and Interfacial Nucleation of H_2_S Hydrates via Molecular Dynamics

**DOI:** 10.1021/acs.jpcb.6c04569

**Published:** 2026-09-25

**Authors:** Joanna Grabowska, Eduardo Sanz

**Affiliations:** † Department of Physical Chemistry, Faculty of Chemistry, 431446 Gdansk University of Technology, ul. Narutowicza 11/12, Gdansk 80-233, Poland; ‡ Dpto. Química Física I, Fac. Ciencias Químicas, 16734 Universidad Complutense de Madrid, Madrid 28040, Spain

## Abstract

Understanding the mechanisms by which gas hydrates nucleate from aqueous solutions is essential for predicting and controlling their formation. Experimental observations have suggested that hydrate nucleation occurs preferentially at interfaces between the aqueous phase and hydrate-forming reservoirs. However, recent simulation studies have raised questions regarding the general validity of this assumption. Here, we investigate the spontaneous nucleation of H_2_S hydrates at 500 bar using molecular dynamics simulations employing the TIP4P/Ice water model and a four-site H_2_S potential, with the aim of clarifying the competition between bulk and interfacial nucleation pathways. To ensure rigorous control of the thermodynamic conditions, we first determine the three-phase coexistence temperature (*T*_3_) from the intersection of the aqueous solubility and hydrate–solution equilibrium curves; our calculated value of *T*_3_ = 314(3) K shows excellent agreement with an experimentally inferred value of approximately 316 K. Then, under deeply supercooled conditions, we directly compare nucleation kinetics across two distinct configurations: an interfacial system with an aqueous solution in contact with an H_2_S reservoir and a standalone bulk solution at identical saturation. Our results show that nucleation rates in bulk systems are comparable to those obtained in the presence of an interface, indicating that the interface does not promote hydrate nucleation under the conditions explored. This behavior is consistent with our previous findings for CO_2_ hydrates and suggests that bulk nucleation can be the prevailing pathway under deep supercooling conditions.

## Graphical abstract

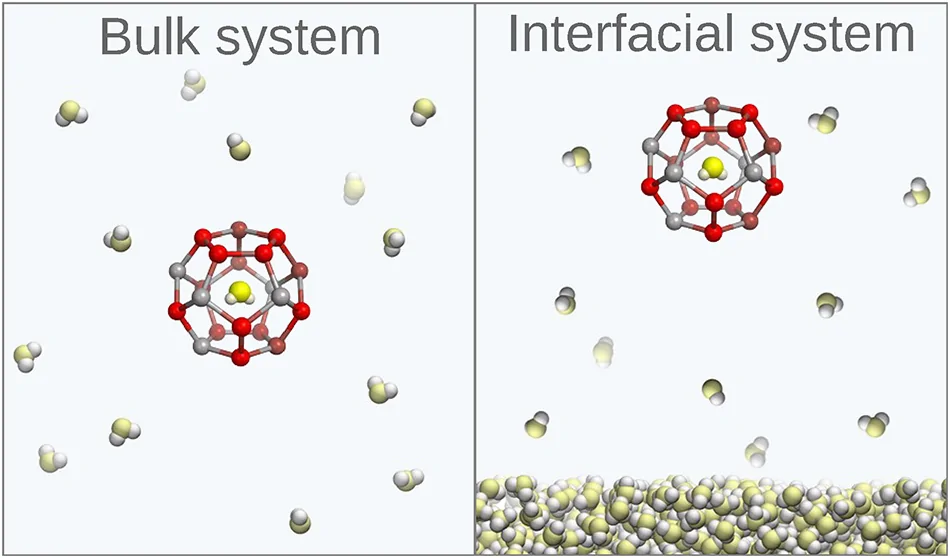

## 1 Introduction

Clathrate hydrates are crystalline solids in which guest gas molecules are encapsulated within hydrogen-bonded water cages.^1^ While widely investigated for potential energy recovery, carbon sequestration, and hydrogen storage,^2–5^ they also pose severe flow-assurance hazards through pipeline plugging during hydrocarbon transport,^6^ making the microscopic mechanisms of hydrate formation and stability of critical importance.

A large number of experimental investigations^7–16^ have been devoted to elucidating the mechanisms governing hydrate formation. Experimental studies have long suggested that hydrate nucleation predominantly occurs at the interface separating the aqueous solution from the guest phase.^1,17–22^ This hypothesis is physically intuitive: the solubility of hydrate-forming gases in water is typically orders of magnitude lower than the guest stoichiometry inside the hydrate lattice, suggesting that the guest-rich interface provides the necessary thermodynamic concentration to overcome the nucleation barrier. However, because of the nanometer dimensions and transient lifetimes of critical nuclei, direct experimental observation of the earliest stages of nucleation remains unfeasible. Consequently, whether the interface genuinely lowers the free-energy barrier for nucleation or simply acts as a guest reservoir feeding dissolved molecules into the boundary layer remains an open debate.^23^

Molecular simulations offer a powerful means to bridge this gap and bring atomistic insight into nucleation phenomena.^24–49^ Moreover, molecular simulations can provide the thermodynamic parameters^50^ required by the theoretical framework describing the competition between different nucleation mechanisms.^23^ Nevertheless, the majority of simulation studies addressing hydrate nucleation have concentrated on homogeneous nucleation in bulk aqueous solutions,^30–35,51–54^ often employing guest concentrations significantly above equilibrium saturation to accelerate nucleation events.

A few recent numerical studies do focus on the location of the nucleation event with respect to that of the interface. Jacobson et al.^55^ observed that nucleation events could occur in the bulk phase or near the interface, whereas a recent study of H_2_S hydrates by Zhang et al.^56^ concluded that an interface is required for nucleation to occur. This contrasts with findings for CO_2_ hydrates,^52,57^ which demonstrated that bulk nucleation can proceed faster than interfacial nucleation under high supercooling. To clarify whether this discrepancy arises from different natures of the hydrate-former molecules (CO_2_ versus H_2_S), we pursue here a study of the nucleation of H_2_S hydrates.

To address this problem, we perform Molecular Dynamics (MD) simulations of spontaneous H_2_S hydrate nucleation in systems containing H_2_S dissolved in water, both in the presence and in the absence of a solution–H_2_S interface. Our simulations resemble experimental conditions in the sense that the H_2_S concentration is given by the equilibrium between the aqueous solutions and the hydrate-former reservoir. Nucleation rates are obtained from direct simulations carried out at sufficiently low temperatures, where the thermodynamic driving force is large enough for nucleation events to occur within simulation times accessible to molecular dynamics (of the order of ∼100 ns). In order to ensure strict control of the thermodynamic conditions explored in this work, we determine, for the first time for this molecular model, the solution–H_2_S–hydrate triple point. This is achieved by computing both the solubility of H_2_S in water and the equilibrium concentration between the aqueous phase and the hydrate phase as functions of temperature, following the methodology described in ref 58. Our results show that, under the studied conditions, the presence of the interface does not enhance hydrate nucleation rates, a behavior consistent with our previous findings for CO_2_ hydrates. These results therefore contribute to the ongoing discussion regarding the dominant nucleation pathway in hydrate-forming systems.

## 2 Methods

### 2.1 Simulation Details

All results presented in this work were obtained using molecular dynamics (MD) simulations. Simulations were carried out with the use of GROMACS.^59,60^ Water was modeled using the TIP4P/Ice^61^ model, a widely used potential, which reliably reproduces the melting temperature and high-pressure volumetric properties of water and ice phases. For H_2_S, a four-site model proposed by Forester et al.^62^ was used. This model was recently used in MD studies of H_2_S hydrate nucleation.^56^ In the literature, the TraPPE model for H_2_S is also available,^63^ but from our testing it seems that this model significantly underestimates the solubility of H_2_S in water under studied conditions (see SI for more details). The dispersive interactions of water and H_2_S were treated with the Lorentz–Berthelot rule.

To assess the volumetric performance of this model combination under elevated pressure, the densities of the coexisting phases were examined. To our knowledge, direct experimental densities for aqueous H_2_S solutions at 500 bar and deep supercooling are unavailable. However, the predicted densities of both pure phases and the aqueous solution are consistent with available reference data. At 260 K and 500 bar, the simulated density of the aqueous phase (with an equilibrium H_2_S mole fraction of ∼6 mol %) is 1017 kg/m^3^, closely mirroring the experimental density of pure liquid water at 500 bar and 273 K (1020 kg/m^3^, NIST).^64^ Furthermore, the density of the pure liquid H_2_S phase at 500 bar and 260 K given by the model—938 kg/m^3^—is consistent with the experimental value at the same temperature but at the vapor pressure, 6.9 bar, of 854 kg/m^3^ if one takes into account the compressing effect of pressure. Moreover, as shown later on, the triple point temperature of the model at 500 bar is consistent with experimental data of hydrate-solution and H_2_S-solution equilibrium data.

The equations of motion were integrated using leapfrog algorithm, with a time step of 2 fs. For temperature and pressure control, Nosé–Hoover thermostat^65,66^ and Parrinello–Rahman barostat^67^ were used, with a relaxation time of 2 ps. A cutoff of 1 nm was used for the dispersive and Coulombic interactions. Particle Mesh Ewald method^68^ was used to compute electrostatic interactions. Long-range corrections for dispersive interactions were not used in simulations.

### 2.2 Construction of Simulation Systems

Three types of systems were prepared: I) bulk systems with a water solution of H_2_S with varying concentrations (*H_2_S­(aq)* from now on), II) two-phase systems where the water solution of H_2_S was in contact with the liquid H_2_S phase via a planar interface (*H_2_S­(l)|H_2_S­(aq)* from now on), and III) two-phase systems where the water solution of H_2_S was in contact with H_2_S hydrate crystal via a planar interface (*H_2_S­(h)|H_2_S­(aq)* from now on). Both kinds of two-phase systems were used for determining the equilibrium concentration of H_2_S in water at various temperatures and at a pressure of 500 bar.

Two-phase systems with liquid H_2_S phase were prepared by placing a pure liquid H_2_S slab in contact with an aqueous H_2_S solution pre-equilibrated at the target temperature. The resulting simulation box had dimensions of 13.2 × 8.5 × 8.5 nm^3^ (with the aqueous phase of 7.5 × 8.5 × 8.5 nm^3^) and contained 5,896 H_2_S molecules and 15,735 water molecules. Each system was initially equilibrated for 50 ps in the *NVT* ensemble at the target temperature within the range 255–420 K. Subsequently, the systems were simulated for at least 200 ns in the Np_*x*_T ensemble—the fluid–fluid interface was oriented perpendicular to the *x* direction and the barostat acted exclusively along the normal direction (*x*) to maintain the target pressure of 500 bar. This choice ensures that the target normal pressure is maintained while strictly preserving a constant, square interfacial area (*A* = *L*_*y*_ × *L*_*z*_), which enhances computational efficiency and eliminates potential artifacts associated with fluctuations of the interfacial geometry. During the equilibration period, the concentration of H_2_S in the aqueous phase reached a stable value equal to the solubility of H_2_S in water (as discussed later on, at low temperatures hydrate nucleation is fast and it precedes equilibration).

Two-phase systems with a hydrate crystal phase were prepared in a similar manner, but with the use of pre-equilibrated H_2_S hydrate crystal instead of H_2_S liquid. The final size of the system was 9.6 × 4.8 × 4.8 nm^3^, where the aqueous solution and the hydrate crystal were occupying the same volume. The composition of the system was 6254 molecules of water and 552 molecules of H_2_S. The equilibration protocol was similar to the *H*_*2*_ *S­(l)|H*_*2*_ *S­(aq)* system: in the first step, the system was simulated in the NVT ensemble for 50 ps, and in the second step, the simulations were conducted in NP_*x*_T ensemble for 400 ns to ensure a proper equilibration of the concentration of H_2_S in the aqueous phase. The systems were simulated under the pressure of 500 bar and at temperatures in the range of 260–320 K.

*H_2_S­(aq)* systems were prepared by inserting molecules of H_2_S into a box filled with liquid water. The number of molecules of H_2_S inserted into the system differed depending on the temperature, so that the molar fraction of H_2_S was equal to the equilibrium solubility of H_2_S in water determined from the simulations of *H_2_S­(l)|H_2_S­(aq)* systems. The final size of the simulation box was 8.5 × 8.5 × 8.5 nm^3^, and the system contained 18319 molecules of water and either 1209 (260 K), 1169 (262 K), or 1118 (265 K) molecules of H_2_S. The equilibration was carried out in two steps: a short 50 ps NVT simulation and a longer isotropic NPT simulation (under the pressure of 500 bar).

## 3 Results and Discussion

### 3.1 The Thermodynamic Foundation: Phase Coexistence and Guest Solubility

A rigorous understanding of hydrate nucleation kinetics requires a precise determination of the underlying thermodynamic driving forces. In this work, we first establish a thermodynamic reference by calculating the equilibrium concentration of H_2_S in water along the 500 bar isobar in two different regimes: in equilibrium with the H_2_S liquid phase and in equilibrium with the H_2_S hydrate phase. The crossing point between the two equilibrium curves is the hydrate-solution-H_2_S triple point, *T*_3_.^58^

As described in Section 2, we prepared *H*_*2*_ *S­(l)|H*_*2*_ *S­(aq)* and *H*_*2*_ *S­(h)|H*_*2*_ *S­(aq)* systems. In both cases, we carried out the simulations using the NP_*x*_T ensemble, with the *x* direction being the direction perpendicular to the interface.

In the case of the *H*_*2*_ *S­(l)|H*_*2*_ *S­(aq)* system, we conducted simulations at temperatures in the range of 250–420 K. The time dependence of the concentration for three representative temperatures is shown in Figure 1A. We start the simulations from an initial configuration with a certain H_2_S concentration in the aqueous phase. During equilibration such concentration varies until it finally reaches a plateau. The average H_2_S concentration in the plateau gives the equilibrium value. At temperatures below *T*_3_, the solid hydrate is more stable than the aqueous solution, and crystallization may occur. In Figure 1A, trajectories are shown in dashed from the moment they contain a growing hydrate nucleus. Such dashed parts of the trajectory exhibit an increasing concentration of H_2_S due to the higher content of H_2_S in the forming hydrate phase relative to the bulk solution. The portion of the trajectory corresponding to the emerging and growing hydrate nucleus was excluded from the equilibrium concentration calculations.

**Figure 1:**
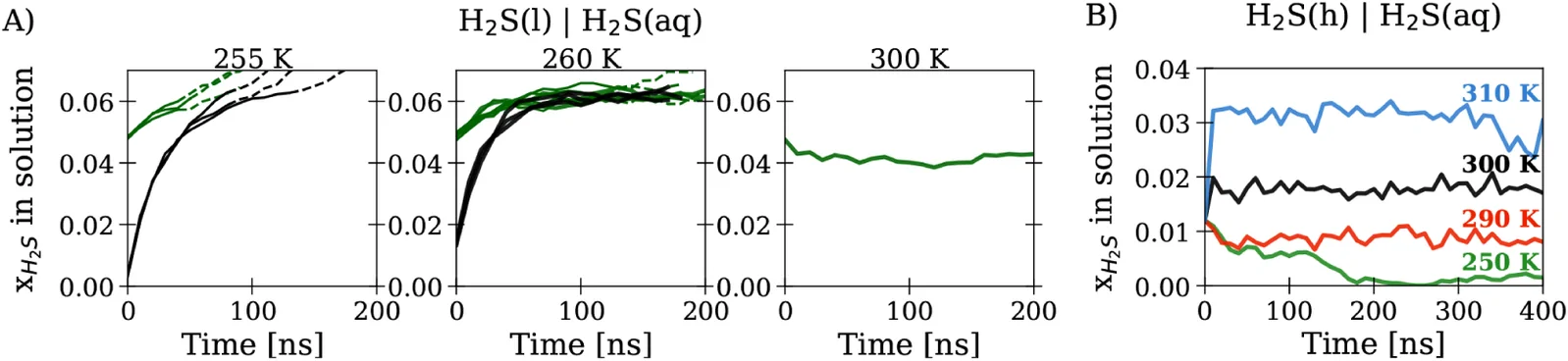
Changes in time of molar fractions of H_2_S in the aqueous phase of two-phase systems where the aqueous solution is in contact with either A) fluid H_2_S phase via a planar interface (*H_2_S­(l)|H_2_S­(aq)* system) or B) H_2_S hydrate phase via a planar interface (*H_2_S­(h)|H_2_S­(aq)* system), at a few selected temperatures. In part A, trajectories corresponding to different starting initial concentrations are shown in different colors (black and green for 0 and ∼0.05 molar fractions, respectively). Also in part A, trajectories are shown in dashed from the moment in which hydrate nucleation is detected.

At temperature 255 K (Figure 1A, left plot) and below, we observed that hydrate nucleation starts very quickly, even before the system has any chance to equilibrate. The first reliable value of the equilibrium solubility of H_2_S in water was obtained at 260 K (Figure 1A, middle plot). In this case, all simulations converge to the same equilibrium concentration, $x_{H_{2}S}\approx 0.06$ , regardless of the initial concentration of H_2_S in the aqueous phase (black and green curves in Figure 1A correspond to different initial concentrations).

Analogous simulations were conducted at higher temperatures of up to 420 K. As an illustrative example, the right plot of Figure 1A depicts the concentration equilibration at 300 K; notably, hydrate nucleation does not occur at these elevated temperatures.

After the systems reached equilibrium (when the concentration of H_2_S in the aqueous phase reached a stable value), we calculated the average molar fraction of H_2_S in the aqueous phase by determining the densities of both H_2_S and water in the bulk part of the system: from 3.5 to 8.0 nm in the *x* direction (see Figure 2, top panel). The results for all temperatures considered in this work are shown in Figure 3 (green dots).

**Figure 2:**
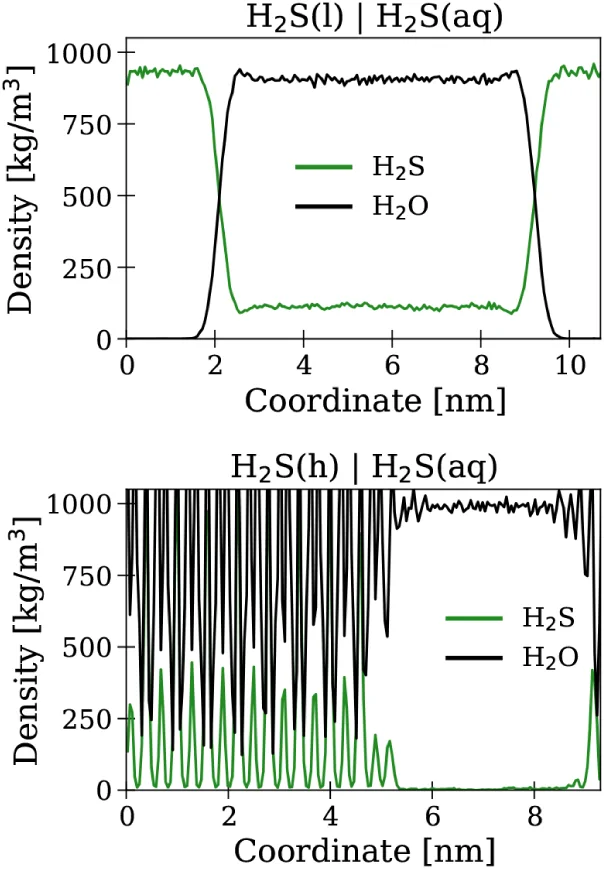
Density profiles of H_2_O (black) and H_2_S (green) for the *H_2_S­(l)|H_2_S­(aq)* (top) and the *H*_2_ *S­(h)|H*_2_ *S­(aq)* systems (bottom) at 260 K, along the *x* direction, which is perpendicular to the interface.

**Figure 3:**
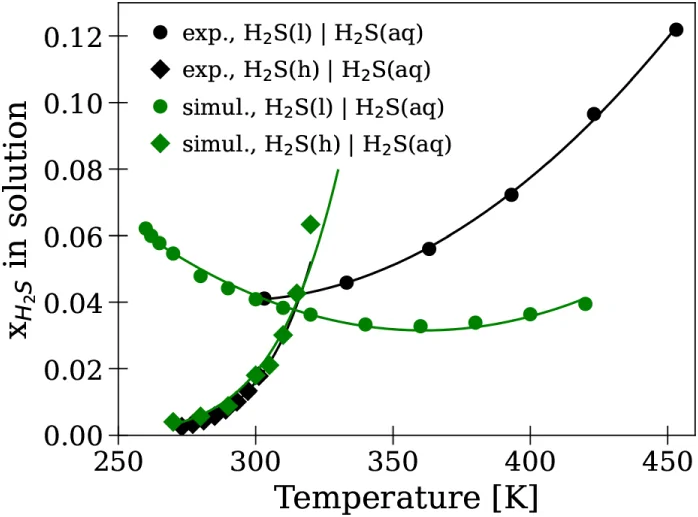
Molar fraction of H_2_S in water when the solution is in equilibrium with the liquid H_2_S phase (dots) and with the H_2_S hydrate (diamonds), at different temperatures and at a pressure of 500 bar. Experimental results^70,71^ are marked in black, and results from simulations are marked in green. Solid lines represent parabolic (*H_2_S­(l)|H_2_S­(aq)*) and exponential (*H_2_S­(h) | H_2_S­(aq)*) fits to the data, respectively, and serve as guides to the eye.

As can be seen in Figure 3, the calculated temperature dependence of the H_2_S concentration has a nonmonotonic shape: starting from the lowest temperature, the solubility of H_2_S in water decreases with increasing temperature until a minimum at ∼360 K, after which an increase of solubility is observed. At the same time, experimental data (black dots in Figure 3) show a monotonous increase of the solubility with increasing temperature. It is worth noting, however, that experimental data are available only for temperatures starting from 300 K, and a seemingly parabolic trajectory of the experimental curve suggests that a solubility minimum may indeed exist at lower temperatures, similar to the behavior captured in our simulations. In fact, the experimental temperature dependence of the solubility of CO_2_ in water does show a minimum under high pressures.^69^ In the case of H_2_S it remains an open question whether the shape of the experimental solubility curve indicates a true parabolic relationship or a more complex functional form where the minimum is shifted or absent in the real system. Given that the solubility of guest molecules in water is highly sensitive to the balance of dispersive interactions and the specific water model employed, these results highlight the necessity for further experimental measurements at deeper supercooling to definitively resolve the low-temperature solubility limit and its impact on the hydrate nucleation driving force.

The results of the H_2_S concentration equilibration in the *H*_*2*_ *S­(h)|H*_*2*_ *S­(aq)* system are presented in Figure 1B (for a few selected runs). At the beginning of each run, the molar fraction of H_2_S in the aqueous phase (calculated in the bulk region of the system, 6.0–8.5 nm in the *x* direction, see Figure 2, bottom panel) was equal to 0.012, and the liquid and solid phases occupied the same volume. During equilibration, depending on the temperature, some of the hydrate melted, causing an increase in the molar fraction of H_2_S in the aqueous solution, or some of the liquid crystallized, resulting in the decrease of the molar fraction of H_2_S in the aqueous phase. Eventually, in all systems, a stable value of molar fraction of H_2_S was observed, which allowed us to calculate the *H*_*2*_ *S­(h)|H*_*2*_ *S­(aq)* equilibrium curve (green diamonds in Figure 3). The temperature dependence of the equilibrium concentration values obtained from our simulations is in a very good agreement with experimental data (see green and black diamonds in Figure 3, respectively). It is also clear that the concentration of H_2_S in the aqueous phase in equilibrium with the hydrate phase increases very rapidly with an increase in temperature. While experimental data provide solubility values up to 300 K, the simulations allow us to obtain results for even higher temperatures (up to 320 K).

The *H*_*2*_ *S­(l)|H*_*2*_ *S­(aq)* and *H*_*2*_ *S­(h)|H*_*2*_ *S­(aq)* equilibrium concentration curves cross at a certain temperature, which defines the three-phase dissociation temperature (T_3_) for the specific force fields used in this work. The estimated value of T_3_ is equal to 314 ± 3 K. To the best of our knowledge, there is no experimental T_3_ reported for real H_2_S hydrates. However, based on the experimental data reported in Figure 3 a T_3_ of about 316 K can be inferred from the crossing between the hydrate-solution^70^ and the H_2_S-solution^71^ equilibrium lines, which is in excellent agreement with the value calculated in this paper. This thermodynamic mapping is crucial, as it allows us to quantify the degree of supercooling at each temperature.

### 3.2 Interfacial vs Homogeneous Nucleation

To test the hypothesis that hydrate nucleation takes place at the interface between the hydrate former and the aqueous solution phases, we conducted simulations at low temperatures seeking for spontaneous nucleation events in bulk aqueous solutions saturated with H_2_S (*H_2_S­(aq)*) and in systems composed of a slab of a H_2_S liquid phase in contact with the aqueous solution (*H_2_S­(l)|H_2_S­(aq)*). Note that the H_2_S concentration in the aqueous solution is the same in both types of systems. If the interfacial nucleation hypothesis is correct, nucleation will occur at a faster rate in the *H_2_S­(l)|H_2_S­(aq)* system. If, on the contrary, the interface does not aid nucleation, both systems will yield similar nucleation rates.

Our first task was to identify a temperature range for which nucleation takes place spontaneously in a simulation lasting a few hundred nanoseconds (an affordable simulation time). Such a temperature range must lie below the triple-point temperature, which is 314 K for the model according to our calculations shown in Figure 3 (below 314 K the saturated aqueous solution is less stable than the solid hydrate).

As discussed in Section 3.1, Figure 1A, left plot, at 255 K crystallization occurs in all trajectories before the concentration reaches an equilibrium value. Consequently, 255 K is not suitable for the study of the crystal nucleation. Furthermore, our simulations reveal that crystallization at this temperature proceeds through multiple nuclei, as shown in Figure 4A. This rapid nucleation at 255 K contrasts with the findings of Zhang et al.,^56^ who used the same H_2_S and H_2_O models and reported an induction period of approximately 100 ns prior to nucleation at the same temperature. This difference may partly arise from the initial concentration of H_2_S in the aqueous phase: in ref 56, the simulations were starting with pure water, requiring additional time for the solution to reach a concentration sufficiently high for nucleation to occur.

**Figure 4:**
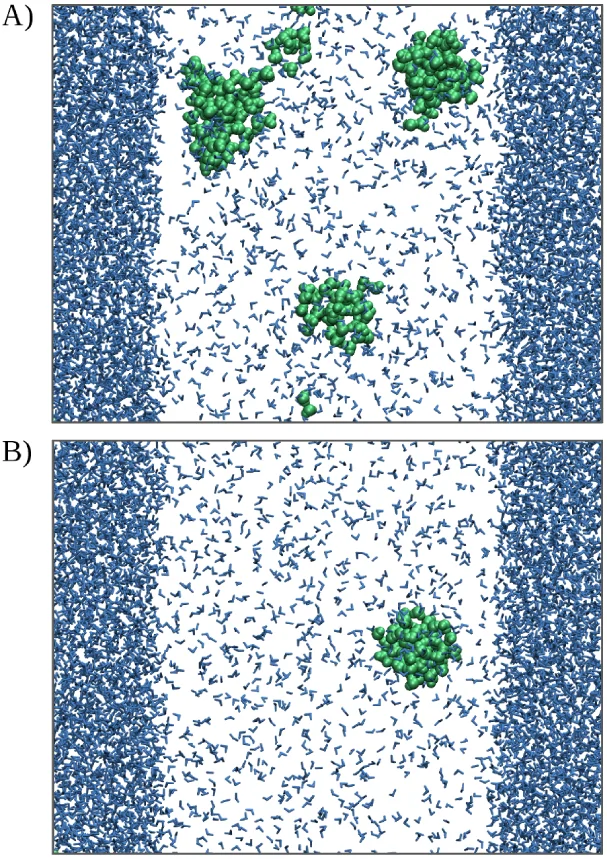
Representative examples of nuclei formed in *H_2_S­(l)|H_2_S­(aq)* systems at A) 255 K and B) 262 K. Legend: Green spheres denote water molecules (oxygen and hydrogen atoms) belonging to the hydrate seed; blue sticks represent H_2_S molecules. Liquid water molecules are omitted for visual clarity. The images were generated using VMD.^72^

Since nucleation at 255 K is too fast, we focus on three higher temperatures, namely 260 K, 262 and 265 K. Figure 5 displays the time dependence of the number of H_2_O molecules in the largest hydrate cluster (identified using the order parameter proposed in ref 52) for these three target temperatures. At 265 K, several trajectories were propagated for both *H*_*2*_ *S­(l)|H*_*2*_ *S­(aq)* and *H*_*2*_ *S­(aq)* systems for one microsecond or longer. Over these time scales, only a single nucleation event was observed, which occurred in the system without interfaces. The very low frequency of nucleation events at 265 K is in contradiction with the ∼500 ns induction period reported for this temperature in ref 56 However, ref 56 considers a system containing a solid-substrate-H_2_S­(aq) interface in addition to the H_2_S­(l)–H_2_S­(aq) interface. Nucleation at the solid-substrate interface could explain the difference in nucleation behavior at 265 K between the present work and that of ref 56.

**Figure 5:**
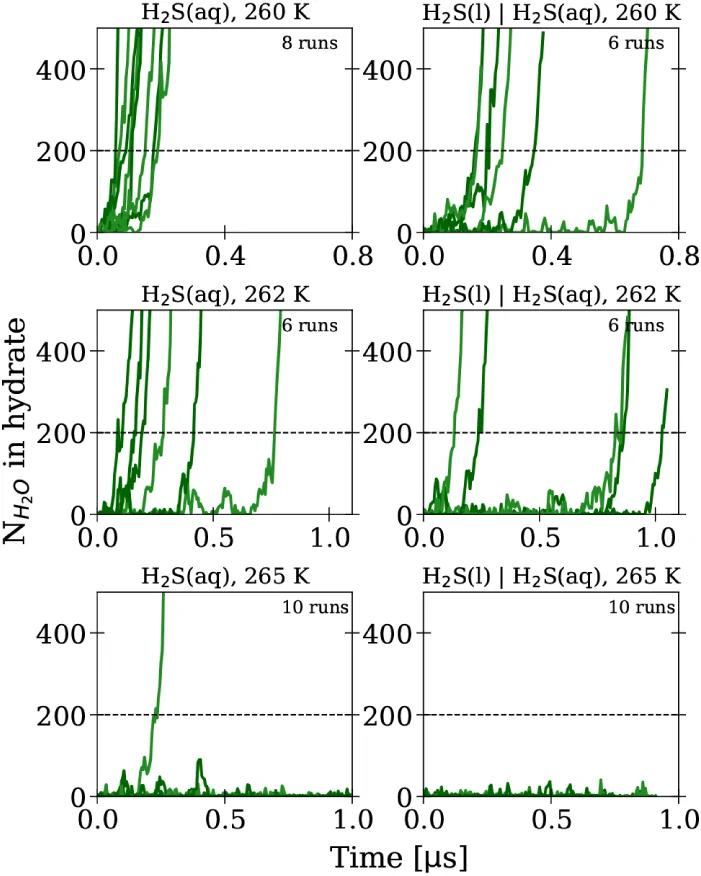
Changes in time of the number of water molecules in the largest cluster of H_2_S hydrate in the bulk *H_2_S­(aq)* system and the two-phase *H_2_S­(l)|H_2_S­(aq)* system at 260, 262, and 265 K.

Having established that nucleation is too rapid at 255 K and too slow at 265 K, we focus on the two intermediate temperatures, 260 and 262 K. To quantify the nucleation kinetics at these temperatures, the nucleation time, *t*_N_, was defined as the time required for the largest hydrate cluster to exceed a threshold of 200 water molecules. This criterion lies well above the subcritical cluster fluctuations characteristic of the induction period and falls within the postcritical, rapid growth regime (see Figure 5). Due to the steep slope of $N_{H_{2}O}$ during this rapid growth phase, the extracted values of *t*_N_ are largely insensitive to the specific choice of threshold, provided it exceeds typical induction-period fluctuations. The resulting nucleation times are summarized in Table 1 for both 260 and 262 K across the bulk and interfacial systems. Note that all reported values exclude the initial 50 ns of the trajectories to account for equilibration of the aqueous H_2_S concentration.

**Table 1:** Average Nucleation Times Obtained for *H_2_S­(aq)* and *H_2_S­(l)|H_2_S­(aq)* Systems, at Temperatures 260 and 262 K^a^

System type	*T*/K	t_*N*, *av*_/ns	V/nm^3^	J/m^–3^s^–1^
*H*_*2*_ *S(aq)*	260	118	604	—
	262	301	604	5 × 10^30^
*H*_*2*_ *S(l)|H*_*2*_ *S(aq)*	260	301	527	—
	262	623	527	3 × 10^30^

a The volume of the aqueous phase and the nucleation rate (at 262 K) are also reported.

The nucleation times obtained in the presence and absence of interfaces are consistent with each other, indicating that interfaces do not promote nucleation. In fact, the nucleation times appear to be systematically longer in the *H*_*2*_ *S­(l)|H*_*2*_ *S­(aq)* system. This trend may be attributed to the slightly smaller size of the interfacial system (∼16k versus ∼18k water molecules in the systems with and without interfaces, respectively). In addition, nucleation could be hindered in the region of the solution adjacent to the interface. This possibility would align with our previous study, where CO_2_ hydrate seeds inserted in close proximity to the interface were less likely to grow than those located further into the bulk phase.^57^ Ultimately, the comparable nucleation times confidently demonstrate that the interface between the solution and the reservoir of the hydrate former does not enhance or promote nucleation under the studied conditions.

For simulations in which a single nucleus emerges following a prolonged induction period, the nucleation rate, *J*, can be defined as:

1 $$J=\frac{1}{V\times \left\langle t_{N}\right\rangle }$$

where *V* denotes the average volume of the aqueous solution and ⟨*t*_*N*_⟩ is the average nucleation time. Eq 1 remains valid under the assumption that only one nucleus forms and propagates, a scenario typical of crystallization occurring after an extended induction period. This behavior is consistently observed at 262 K (see Figure 5). In fact, Figure 4B illustrates the growing *single* nucleus within a representative trajectory at this temperature. Conversely, eq 1 cannot be applied to the simulations at 260 K, as the induction period is too short and several trajectories exhibit multiple nucleation events. At 262 K, the calculated nucleation rates are on the order of 10^30^ m^–3^ s^–1^ for both the *H*_*2*_ *S­(l)|H*_*2*_ *S­(aq)* and *H*_*2*_ *S­(aq)* systems (see Table 1), further confirming that the interface does not influence the nucleation frequency.

To further verify that nucleation is not assisted by the interface, we tracked the spatial origin of the emerging nuclei within the *H_2_S­(l)|H_2_S­(aq)* system. Specifically, the center-of-mass coordinate of the largest hydrate cluster was computed along the axis perpendicular to the interface and plotted as a function of time, as shown in Figure 6. Dashed horizontal lines indicate the interfacial region, where the H_2_S concentration varies from the bulk *H_2_S­(l)* value (indicated in black) to that of the bulk *H_2_S­(aq)* phase (indicated in green). The region bounded by the green lines contains the aqueous solution, characterized by a homogeneous density and composition (matching the conditions maintained in the bulk *H_2_S­(aq)* system against which the nucleation behavior in the *H_2_S­(l)|H_2_S­(aq)* system is compared). Two representative, independent nucleation trajectories are displayed in separate panels, with symbols colored according to the cluster size. The vertical lines denote the effective cluster diameter, which was calculated from the number of H_2_O molecules in the cluster by assuming a spherical geometry and bulk hydrate density. In both cases (as well as in other analyzed trajectories not shown), the clusters emerge strictly within the bulklike region of the aqueous phase. As illustrated in the lower panel, the growing cluster eventually contacts the interface, however, the critical nucleation event clearly takes place within the bulk region. This spatial analysis, combined with the comparable nucleation times observed between bulk and interfacial setups, strongly corroborates that nucleation is not interface-driven under the investigated conditions.

**Figure 6:**
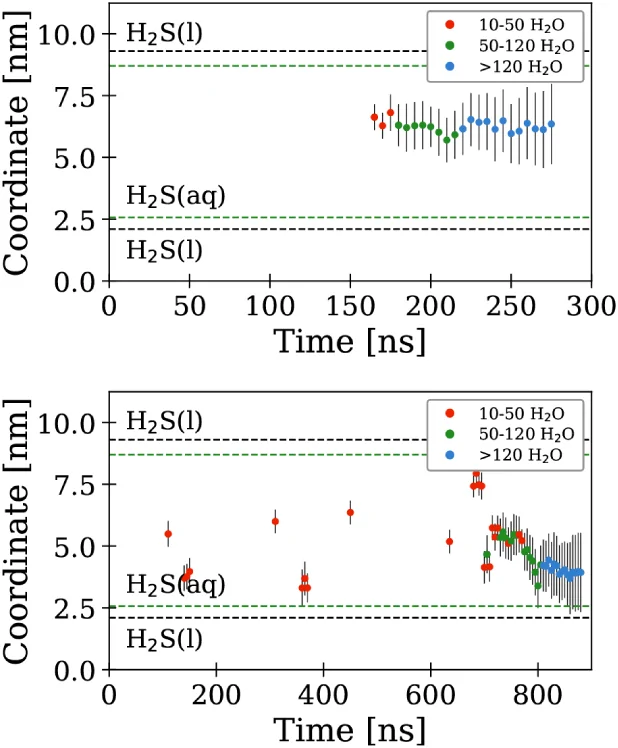
Center-of-mass position of the largest hydrate cluster along the direction normal to the interface as a function of time, presented for two representative trajectories at 262 K. Colors indicate the cluster size in terms of the number of water molecules (see legend). Vertical bars represent the estimated cluster diameter, assuming a spherical cluster with the density of bulk hydrate. Consecutive points separated by 5 ns do not necessarily correspond to the same cluster. Dashed horizontal lines mark the interfacial regions. The H_2_S concentration in the aqueous phase is homogeneous between the green lines.

## 4 Conclusions

In this work, we investigated the nucleation pathway of H_2_S hydrates at 500 bar using molecular dynamics simulations. By employing the TIP4P/Ice^61^ potential for water alongside the four-site model for H_2_S developed by Forester et al.^62^ we first successfully determined the three-phase coexistence temperature, *T*_3_, from the intersection of the hydrate–solution and H_2_S–solution equilibrium curves. Our calculated value of *T*_3_ = 314(3) K is in very good accordance with the inferred experimental value of 316 K, validating the thermodynamic accuracy of our simulation framework. Utilizing this validated model under deeply supercooled conditions, we performed extended simulations across two distinct configurations: an interfacial system where the aqueous solution is in contact with a liquid H_2_S reservoir and a standalone bulk solution at identical saturation—to elucidate the precise role of the fluid–fluid interface during spontaneous hydrate nucleation.

By comparing these two systems, we systematically evaluated whether the presence of a fluid–fluid interface alters the nucleation mechanism or kinetics. In both setups, spontaneous nucleation was observed only under deep supercooling. Our results demonstrate that the nucleation behavior is remarkably similar across both configurations at all temperatures investigated. At 255 K, crystallization proceeds rapidly, even before the concentration reaches an equilibrium value, via the simultaneous formation and growth of multiple nuclei. At 260 K, we find a mix of both single- and multinuclei trajectories. Nucleation at this temperature starts after the concentration is well equilibrated. Upon raising the temperature to 262 K, all trajectories exhibit a single, well-defined nucleation event, enabling a robust estimation of the nucleation rate. Crucially, the nucleation rates calculated for the interfacial and bulk setups are identical within statistical uncertainty. This finding provides direct evidence that the fluid–fluid interface does not enhance or catalyze hydrate nucleation under the studied conditions. Moreover, we analyze the birth location of nuclei in the interfacial system. In all cases, nuclei appear in the bulk-like region of the aqueous solution, far away from the interface.

The absence of interfacial acceleration during H_2_S hydrate nucleation is consistent with our previous simulations on CO_2_ hydrate systems,^52^ yet it contrasts sharply with a recent simulation study^56^ suggesting that H_2_S nucleation does not occur within the bulk aqueous phase. Experimentally, it is frequently assumed that hydrate nucleation takes place preferentially at the fluid–fluid interface.^15,19^ However, our findings demonstrate that, at least under the deeply supercooled conditions investigated here, such an interface is not a prerequisite for nucleation. While these insights clarify the intrinsic bulk-like nature of the nucleation event, further systematic studies remain essential to fully understand the interfacial influence across varying driving forces and different guest molecules.

The present findings leave open critical questions regarding hydrate nucleation under moderate driving forces closer to the three-phase coexistence temperature (*T*_3_), where homogeneous nucleation is kinetically inaccessible within simulation time scales. Under such low supercoolings, it remains possible that interfacial pathways become energetically favorable compared to the bulk or that heterogeneous mechanisms driven by impurities or solid surfaces play a decisive role in triggering hydrate formation. Addressing these regimes represents a natural and essential continuation of the research trajectory initiated in our previous studies on CO_2_ systems^52^ and extended here to H_2_S hydrates.

## Supplementary Material



## Notes

Comparison of the equilibrium solubility of H_2_S in water at various temperatures and at 500 bar, obtained using two different models of H_2_S: the model proposed by Forester et al.^62^ and the TraPPE model^63^ (PDF)
