# Supplementary material for: Assessing the Competition between Bulk and Interfacial Nucleation of H2S Hydrates via Molecular Dynamics
Source: J Phys Chem B. 2026 Sep 25;130(40):10601–8. doi: 10.1021/acs.jpcb.6c04569 (PMC13656768; doi:10.1021/acs.jpcb.6c04569)
Supplement: Supplementary file 1 [file jp6c04569_si_001.pdf]

Supporting Information

Assessing the Competition Between Bulk and Interfacial Nucleation of H_2S Hydrates via Molecular Dynamics

Joanna Grabowska*,† and Eduardo Sanz*,‡

†*Department of Physical Chemistry, Faculty of Chemistry, Gdansk University of Technology, ul. Narutowicza 11/12, 80-233 Gdansk, Poland*

‡*Dpto. Química Física I, Fac. Ciencias Químicas, Universidad Complutense de Madrid, 28040 Madrid, Spain*

E-mail: joanna.grabowska@pg.edu.pl; esa01@quim.ucm.es

Comparison of the solubilities of H_2S in water obtained with two different H_2S models - model proposed by Forester et al.[1] and TraPPE[2] model.

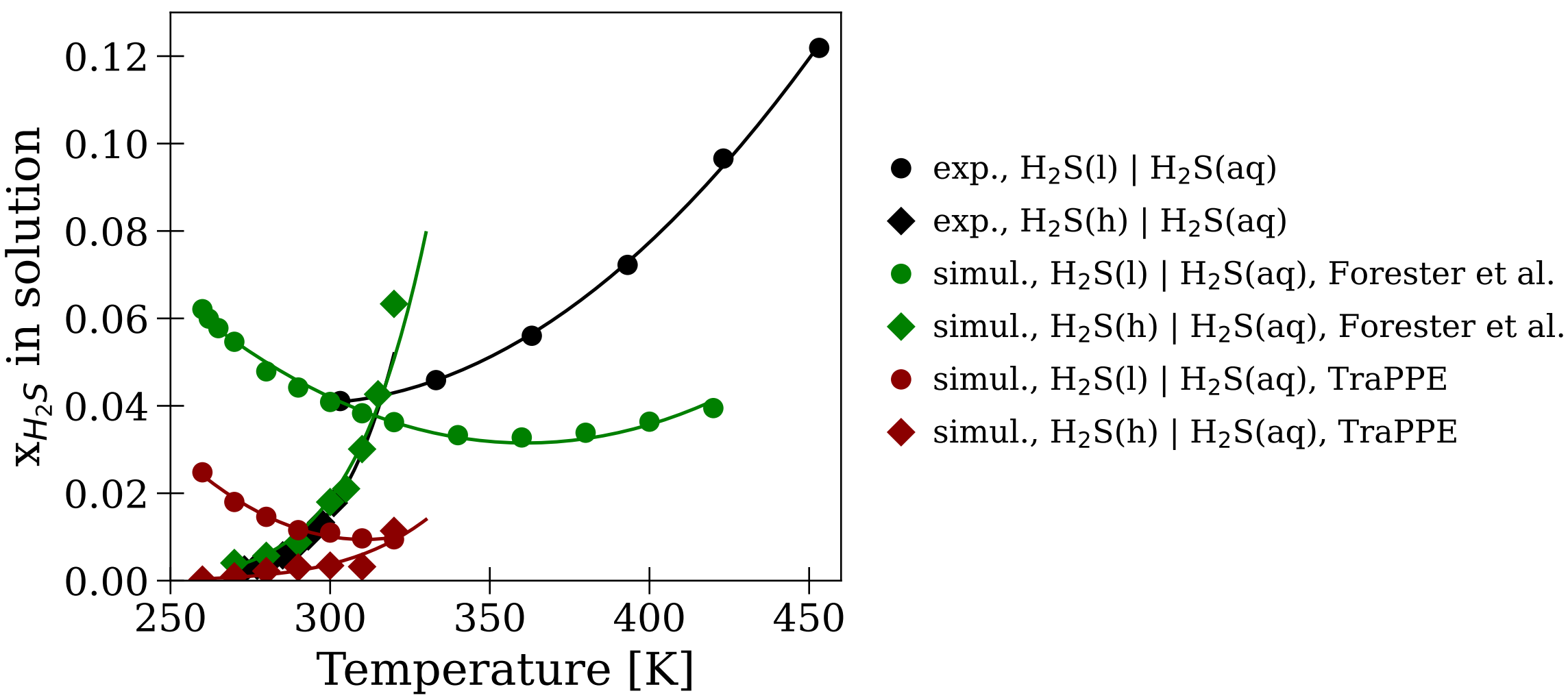

Figure S1: Equilibrium solubilities of H_2S in water from liquid H_2S phase (dots) and H_2S hydrate (diamonds), at various temperatures and pressure of 500 bar. Experimental results are marked in black, results from simulations using the model proposed by Forester et al.[1] are marked in green, and results from simulations using the TraPPE[2] model (4-3 type) are marked in red.

References

(1) Forester, T.; McDonald, I.; Klein, M. Intermolecular potentials and the properties of liquid and solid hydrogen sulphide. *Chemical Physics* **1989**, *129*, 225–234.

(2) Shah, M. S.; Tsapatsis, M.; Siepmann, J. I. Development of the Transferable Potentials for Phase Equilibria Model for Hydrogen Sulfide. *The Journal of Physical Chemistry B* **2015**, *119*, 7041–7052.
